# Weak representation of awake/sleep states by local field potentials in aged mice

**DOI:** 10.1038/s41598-022-11888-0

**Published:** 2022-05-11

**Authors:** Daichi Konno, Yuji Ikegaya, Takuya Sasaki

**Affiliations:** 1 Laboratory of Chemical Pharmacology, Graduate School of Pharmaceutical Sciences, The University of Tokyo, Tokyo, 113-0033 Japan; 2 Laboratory of Geriatric Medicine, Graduate School of Medicine, The University of Tokyo, Tokyo, 113-0033 Japan; 3 Institute for AI and Beyond, The University of Tokyo, Tokyo, 113-0033 Japan; 4 Center for Information and Neural Networks, National Institute of Information and Communications Technology, Suita City, Osaka 565-0871 Japan; 5 Department of Pharmacology, Graduate School of Pharmaceutical Sciences, Tohoku University, 6-3 Aramaki-Aoba, Aoba-Ku, Sendai, 980-8578 Japan

**Keywords:** Neuroscience, Neural ageing

## Abstract

Senescence affects various aspects of sleep, and it remains unclear how sleep-related neuronal network activity is altered by senescence. Here, we recorded local field potential signals from multiple brain regions covering the forebrain in young (10-week-old) and aged (2-year-old) mice. Interregional LFP correlations across these brain regions could not detect pronounced differences between awake and sleep states in both young and aged mice. Multivariate analyses with machine learning algorithms with uniform manifold approximation and projection and robust continuous clustering demonstrated that LFP correlational patterns at multiple frequency bands, ranging from delta to high gamma bands, in aged mice less represented awake/sleep states than those in young mice. By housing aged mice in an enriched environment, the LFP patterns were changed to more precisely represent awake/sleep states. Our results demonstrate senescence-induced changes in neuronal activity at the network level and provide insight into the prevention of pathological symptoms associated with sleep disturbance in senescence.

## Introduction

Senescence reduces the quality of sleep (e.g., shorter sleep time, longer sleep-onset latency, sleep fragmentation, and insomnia)^1–3^. Sleep disturbance in senescence increases the onset risk of many diseases, such as Alzheimer's disease, depression, and diabetes^4^. Many biological factors at the molecular and cellular levels in the aged brain have been revealed, such as the loss of neurotransmitters^5^, synapses^6,7^, and neurons^8^. The integration of these microscopic mechanisms is considered to reduce brain volume^9–12^ and disrupt neuronal network activity that sustains normal awake/sleep cycles.

Physiological studies have demonstrated that cortical brain oscillations in specific frequency bands are reduced by aging^13–16^. These observations suggest that neuronal network mechanisms to create sleep-related oscillatory signals are degraded in senescence. To further understand sleep-related brain network activity in senescence, functional connectivity (i.e., correlated activity) patterns across multiple brain regions need to be considered. As sleep states are maintained through interactions with many, not single, brain regions in various frequency bands^17^, investigations from multiple brain areas and multiple frequency bands are necessary to comprehend senescence-related neuronal network activity. In addition, investigations with temporal windows associated with dynamic changes between awake/sleep states, rather than entire data sampling periods, are necessary to precisely capture and evaluate senescence-related neuronal network activity.

To address these issues, this study compared awake/sleep-related neuronal network activity between young (10 week) and aged (2 year) mice. The 2-year mice were selected as aged mice based on a report that the average lifespan of C57/B6J mice is 2.37 years^18^. Correlational and multivariate analyses were applied to local field potential (LFP) data recorded from multiple brain regions covering the entire forebrain area. After identifying decreases in dynamic changes in LFP signals that differentiate awake/sleep states in aged mice, we assessed whether such awake/sleep-related brain activity can be changed by housing them in an enriched environment (EE).

## Results

### LFP recordings from multiple brain regions in awake/sleep states in aged mice

**Figure 1:**
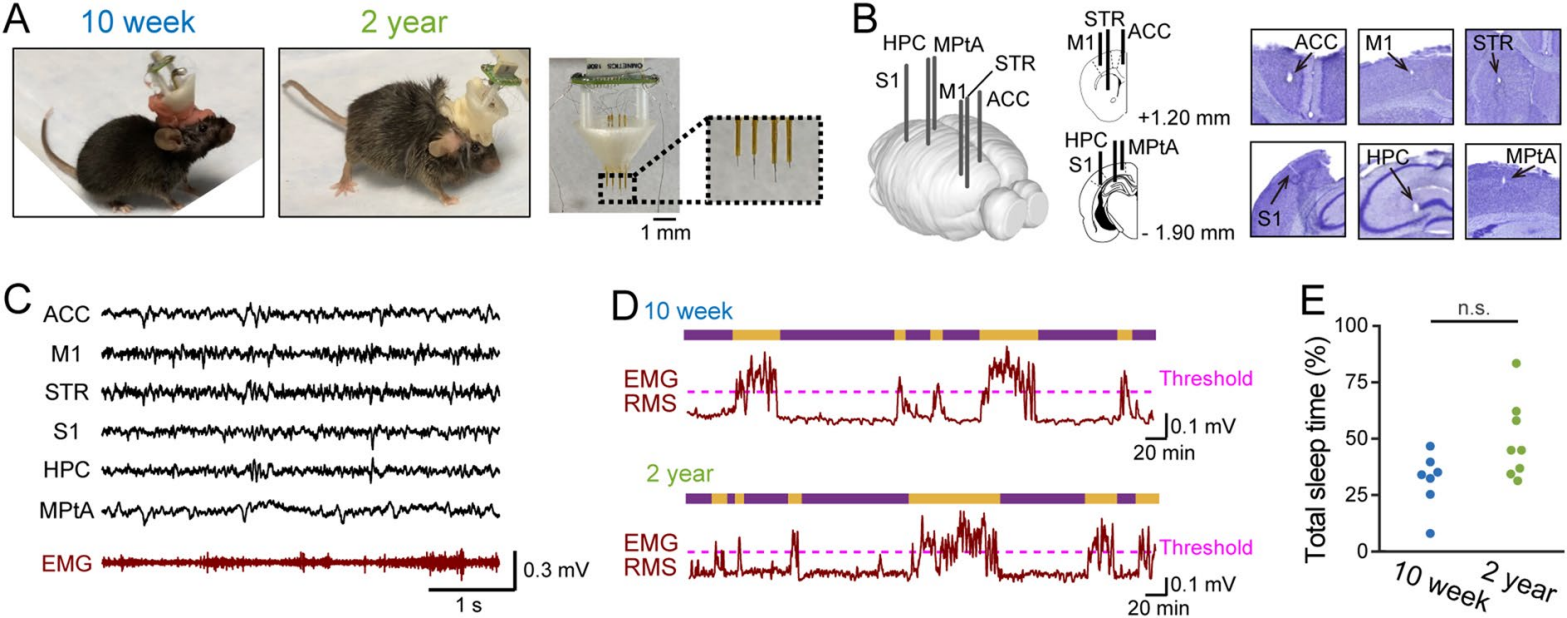
*Multisite LFP recordings and definition of sleep states in aged mice*. (**A**) (Left) A 10-week and 2-year mouse implanted with an electrode array. (Right) A custom-made electrode array. The dotted box is magnified in the right inset, showing electrodes protruding from the electrode assembly with a length of 0.5–2.3 mm to be inserted into the brain. (**B**) (Left) Electrodes implanted into six brain regions. (Right) Histological confirmation of an electrode in each region. (**C**) Representative LFP signals recorded from the six brain regions (black) and an EMG signal (brown). (**D**) The RMS (magnitude) of EMG signals in 10-week (top) and 2-year (bottom) mice. The magenta dotted lines represent the detection thresholds. The upper bars represent sleep (purple) and awake (orange) states. (**E**) The percentage of sleep periods defined from EMG signals to total recording periods (*n* = 7 and 8 mice). Each dot represents an individual mouse. *P < 0.05, Mann–Whitney U test. MATLAB R2021b (https://jp.mathworks.com/products/new_products/release2021b.html) and Illustrator 26.2.1 (https://www.adobe.com/jp/products/illustrator.html) were used to create this figure.

We established a recording system that simultaneously monitors LFP signals from six brain regions and dorsal neck EMG signals in freely moving mice using a custom-made plastic device (Fig. 1A). The six brain regions included the anterior cingulate cortex (ACC), primary motor cortex (M1), striatum (STR), primary somatosensory cortex (S1), hippocampus (HPC), and medial parietal association cortex (MPtA) (Fig. 1B). These brain areas were selected so that they covered the wide range of the forebrain from anterior to posterior parts. Recording sites were verified by histological inspection. Figure 1C shows representative simultaneous recordings of LFP traces and an EMG trace obtained from a freely moving mouse. These electrophysiological signals were recorded from young (8- to 10-week-old; collectively termed 10-week) and aged (95- to 105-week-old; collectively termed 2-year) mouse groups. All recordings were performed in the familiar home cage that was transported to a novel soundproof room. Therefore, the mice were acclimated to recording environments. For each mouse, a recording time was 2.5–3.5 h.

First, we classified all recording periods into awake/sleep states based on the combination of EMG signals and cortical LFP signals (Supplementary Fig. S1A). EMG signals were converted to root mean square (RMS) traces (Fig. 1D) to define active awake states with a threshold of mean + 0.8–1.2 × SD (Supplementary Fig. 1C, top raw), which represents apparent animal’s movement (Supplementary Fig. 1B). In addition, according to the awake/sleep classification utilized in a previous study ^19–24^. we defined slow wave sleep (SWS) states (Supplementary Fig. 1C, middle raw), rapid eye movement (REM) sleep states (Supplementary Fig. 1C, bottom raw), and quiet awake states, based on the threshold defined by delta and theta power of cortical LFP signals and the EMG RMS traces (Fig. 1D; for more detail, see Methods). For further analyses, active awake and quiet awake states were summarized as awake periods, whereas SWS and REM sleep states were summarized as sleep periods. In the representative 10-week and 2-year mice shown in Fig. 1D, sleep time accounted for 30.1% and 44.9% of the total recording time, respectively (individual data shown in Supplementary Fig. 1D). Overall, the percentage of sleep time in 2-year mice was not significantly different from that in 10-week mice (Fig. 1E; *n* = 7 and 8 mice; *Z* = 1.79, *P* = 0.073, Mann–Whitney U test).

To confirm that our recording conditions were stable over time and not strongly affected by time-dependent effects (e.g. acclimatation over time), we compared sleep time observed from the first 20 min and the last 20 min of the recording period (Supplementary Fig. 1E). No significant differences were found between the two time periods in both the 10-week (*n* = 7 mice; *Z* = 0.67, *P* = 0.49, Wilcoxon signed rank test) and 2-year-old groups (*n* = 8 mice; *Z* = 1.40, *P* = 0.17, Wilcoxon signed rank test). These results verify that the frequency of awake/sleep cycles did not prominently change over the entire recording time.

### No pronounced differences in interregional LFP correlations between awake and sleep states both in young and aged mice

**Figure 2:**
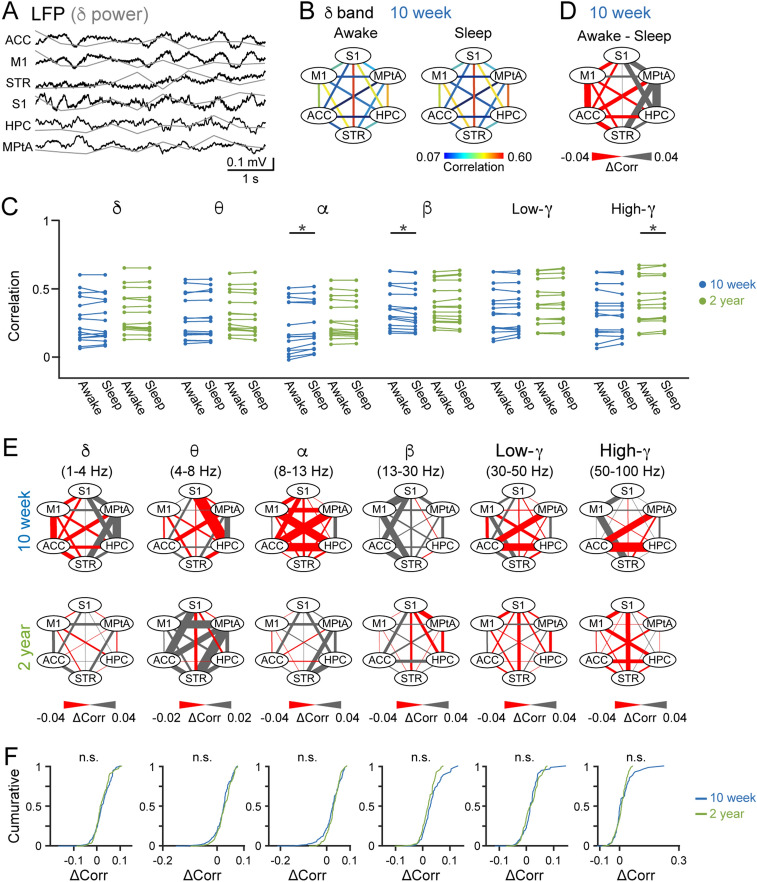
*Interregional correlations of LFP power changes across awake/sleep states in young and aged mice*. (**A**) LFP signals were converted into normalized delta power (superimposed as gray lines) every 1 s. (**B**) Representative color-coded maps showing correlation coefficients of delta power changes for 15 pairs of the six brain regions. Data are from a representative 10-week mouse. (**C**) Comparisons of averaged correlational power changes in six frequency bands between awake and sleep states. Each line represents each brain region pair. *P < 0.05, Wilcoxon signed-rank test followed by Bonferroni correction. (**D**) A color-coded map showing differences in the power correlations (Δcorr) between awake and sleep states, constructed from B. (**E**) Color-coded maps showing Δcorr in awake and sleep states in six frequency bands. Data are averaged from all mice. (**F**) Cumulative distributions of Δcorr. *P* > 0.05, Mann–Whitney U test. MATLAB R2021b (https://jp.mathworks.com/products/new_products/release2021b.html) and Illustrator 26.2.1 (https://www.adobe.com/jp/products/illustrator.html) were used to create this figure.

We next analyzed LFP signals from multiple brain regions. For each brain region, an LFP signal was converted to its power traces in six frequency bands (delta (δ), 1–4 Hz; theta (θ), 4–8 Hz; alpha (α), 8–13 Hz; beta (β), 13–30 Hz; low-gamma (low-γ (L)), 30–50 Hz; high-gamma (high-γ (H)), 50–100 Hz) (Fig. 2A). First, we compared LFP power in these individual frequency bands averaged over all awake and sleep states. Several brain regions showed differences in LFP power at a single frequency between awake and sleep states in both 10-week and 2-year mice (Supplementary Fig. 2A). In all brain regions, delta power was significantly higher during SWS states, compared with awake states (Supplementary Fig. 2B), consistent with previous studies^19,25^.

We next examined regional correlations of LFP power changes (i.e., functional connection) in awake/sleep states. To obtain an overview of functional connections from all datasets, we computed correlation coefficients of LFP power changes in individual frequency bands between two brain regions throughout the entire recording session. Correlation coefficients from all brain region pairs (_6_C_2_ = 15) from a representative mouse are summarized as a color-coded map and shown in Fig. 2B. In both the 10-week and 2-year mice, the correlation coefficients in all the pairs were not significantly different between awake and sleep states in the majority of frequency bands, except few frequency bands (Fig. 2C; *n* = 7 and 8 mice; *P* > 0.99 (10-week, delta), *P* > 0.99 (10-week, theta), *P* = 0.032 (10-week, alpha), *P* = 0.0036 (10-week, beta), *P* > 0.99 (10-week, low-gamma), *P* > 0.99 (10-week, high-gamma), *P* > 0.99 (2-year, delta), *P* = 0.051 (2-year, theta), *P* = 0.29 (2-year, alpha), *P* > 0.99 (2-year, beta), *P* = 0.33 (2-year, low-gamma), *P* = 0.00090 (2-year, high-gamma), Wilcoxon signed rank test followed by Bonferroni correction). Next, we compared awake/sleep differences between the 10-week group and the 2-year group by calculating differences in correlation coefficients between awake and sleep states as Δcorr in individual brain region pairs and summarized as a color-coded map (Fig. 2D). Figure 2E shows all maps of Δcorr in the six frequency bands averaged over all mice. Overall, cumulative distributions revealed no significant differences in Δcorr between the 10-week group and the 2-year group in all frequency bands (Fig. 2F; *n* = 7 and 8 mice; *P* > 0.99 (delta), *P* > 0.99 (theta), *P* = 0.22 (alpha), *P* > 0.99 (beta), *P* > 0.99 (low-gamma), *P* > 0.99 (high-gamma), Mann–Whitney U test followed by Bonferroni correction). These results demonstrate that LFP power changes at single frequency bands at single brain region pairs are not sufficient to differentiate awake and sleep states both in young and aged mice.

### Weak representation of awake/sleep states by LFP patterns at multiple frequency bands in aged mice

**Figure 3:**
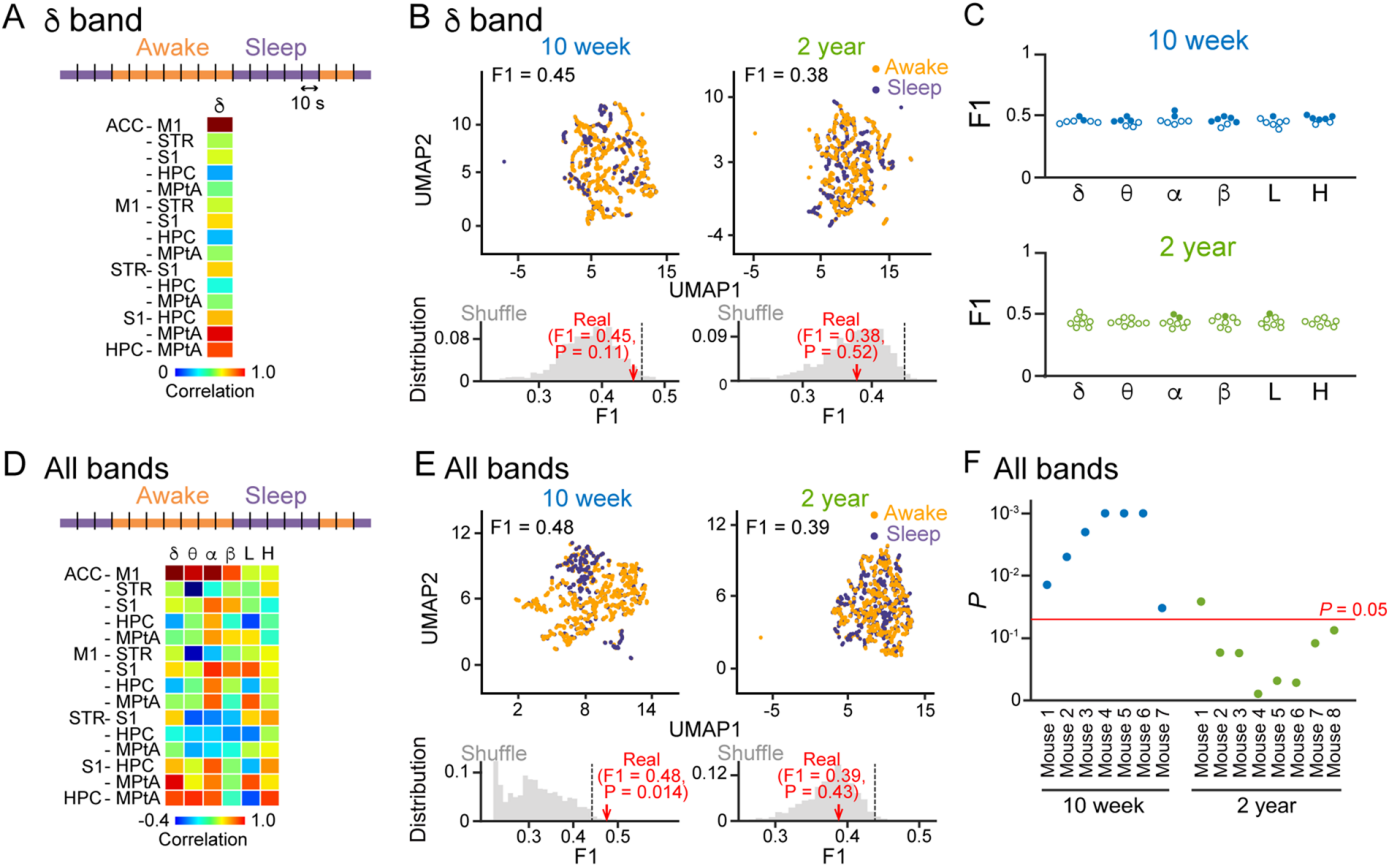
*LFP correlational patterns less represented awake and sleep states in aged mice*. (**A**) Schematic of analysis. Each 10-s bin was categorized as an awake (orange) or a sleep (purple) bin based on EMG signals, and correlations of power changes in a frequency band across the 15 region pairs were concatenated in a column. (**B**) (Top) Visualization of single-mouse data by UMAP plots. Orange and purple dots represent awake and sleep 10-s bins, respectively. F1 scores to quantify the separation of awake/sleep states from LFP patterns are shown above. (Bottom) The F1 score shown in the top panel (real, red arrow) was compared with a distribution of F1 scores computed from 1000 shuffled datasets in which awake/sleep states of all the plots were randomly shuffled (gray distribution). For each original F1 score, its significance was represented as a *P* value defined from the ranking compared with surrogate F1 scores. (**C**) F1 scores computed in individual frequency bands. Each dot represents an individual mouse. The closed and open circles represent significant and nonsignificant data, respectively. (**D**,**E**) Same as A and B, but correlations in the six frequency bands were used for the UMAP analysis. (**F**) For individual mice, *P* values for original F1 scores were computed from datasets including all six frequency bands. The horizontal red line indicates *P* = 0.05, showing the significance. MATLAB R2021b (https://jp.mathworks.com/products/new_products/release2021b.html), Illustrator 26.2.1 (https://www.adobe.com/jp/products/illustrator.html) and Python 3.10.4 (https://www.python.org/downloads/release/python-3104/) were used to create this figure.

The analyses in Fig. 2 focused on LFP signals at single frequency bands averaged over all awake/sleep periods. Under natural conditions, awake/sleep states continuously vary across time (generally tens of seconds to several minutes). We next asked whether such time-varying awake/sleep state patterns could be differentiated by instantaneous brain LFP patterns. To this end, recording periods of 2.5–3.5 h were divided into bins of 10 s, and each bin was categorized as an awake or a sleep bin (for more detail, see Methods). In each bin, correlation coefficients of LFP power changes at each frequency band were computed across the 15 pairs of the six brain regions to obtain a 15-dimensional vector (Fig. 3A). Our analysis tested whether these correlational LFP patterns in each bin could represent an awake or sleep bin. For each frequency band, we applied UMAP (uniform manifold approximation and projection), a dimension reduction technique without any subjective bias, to these vectors. Figure 3B shows representative two-dimensional UMAP plots in the delta band for a 10-week and 2-year mouse. In these graphs, the plots appeared to be not clearly separated between awake and sleep bins. To quantify the degree of separation, the robust continuous clustering (RCC) algorithm was applied to all the plots in each graph. After identifying clusters by the RCC, each cluster was categorized as an awake or a sleep cluster, depending on whether there were more awake or sleep bins in the cluster, respectively. An F1 score was then computed as an index based on the percentage of awake/sleep bins assigned in awake/sleep clusters (Fig. 3B,C). To assess the significance of an F1 score, shuffled datasets were constructed by randomly shuffling types (awake/sleep) to which the individual plots across all the plots were categorized. An F1 score was considered to be significant when it was higher than the top 5% of F1 scores computed from 1000 shuffled datasets (a distribution shown in the bottom panel in Fig. 3B). Of the seven 10-week mice, five (71.4%), three (42.8%), five (71.4%), two (28.6%), six (85.7%), and two (28.6%) animals did not show significant F1 scores for the delta, theta, alpha, beta, low-gamma, and high-gamma bands, respectively (Fig. 3C; indicated by the open circles). Of the eight 2-year mice, eight (100%), eight (100%), six (75.0%), seven (87.5%), seven (87.5%), and eight (100%) animals did not show significant F1 scores for the six frequency bands (Fig. 3C; indicated by the open circles). These results suggest that regional correlations of LFP power changes computed in a single frequency band do not perfectly represent awake/sleep states in both of the ages.

Next, we asked whether the awake/sleep separations in LFP patterns were improved when the datasets in all six frequency bands were employed in an analysis. All vectors in the six frequency bands in individual bins were combined to construct 90-dimensional vectors (Fig. 3D), and the same procedures, plotting on UMAP dimensions and computing F1 scores, were applied to these 90-dimensional vectors (Fig. 3E). All 10-week mice showed significant F1 scores, whereas only 12.5% (1/8) of the 2-year mice showed significant F1 scores (Fig. 3F). Taken together, our multidimensional analyses demonstrate that awake and sleep states are less precisely represented by LFP correlational patterns representing entire brain networks in 2-year mice than in 10-week mice.

### Enriched environment restores LFP correlations to represent awake/sleep states in aged mice

**Figure 4:**
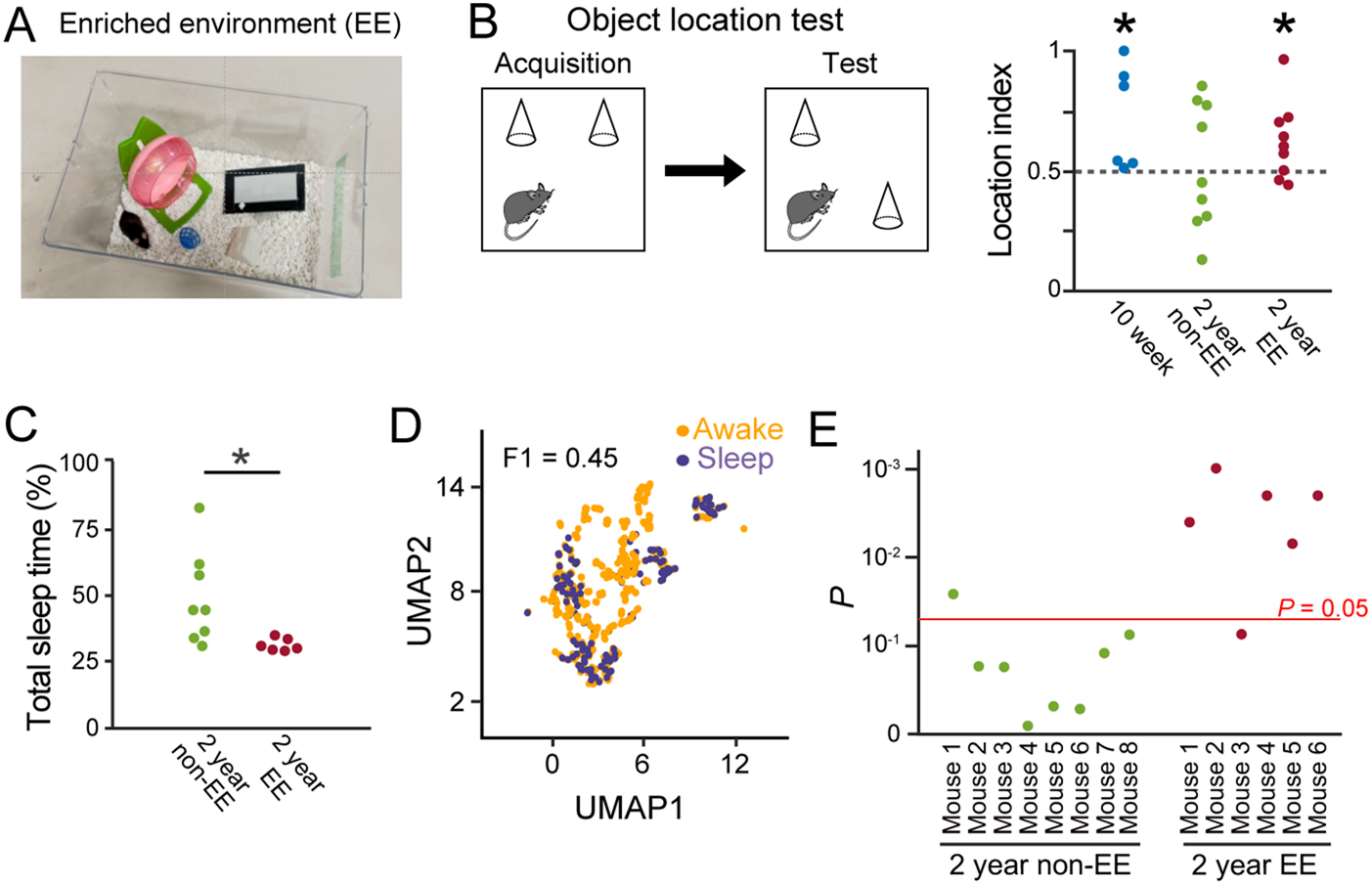
*EE restores sleep-related neuronal activity in aged mice*. (**A**) A photograph of the EE. (**B**) (Left) Schematic diagram of an object location test. (Right) Location index in the object location test (*n* = 6, 9, and 9 mice). Each dot represents an individual mouse. *P < 0.05, Wilcoxon signed rank test versus 0.5 (dotted line). (**C**) The percentage of sleep periods to total recording periods (*n* = 7, 8, and 6 mice). The plots in the 2-year non-EE groups are similar to those shown in Fig. 1e, presented for comparison. *P < 0.05, Mann–Whitney U test. (**D**) Same as Fig. 3e but for a representative 2-year EE mouse. (**E**) For individual mice, *P* values for original F1 scores were computed from datasets including all six frequency bands. The horizontal red line indicates *P* = 0.05, showing the significance. MATLAB R2021b (https://jp.mathworks.com/products/new_products/release2021b.html), Illustrator 26.2.1 (https://www.adobe.com/jp/products/illustrator.html) and Python 3.10.4 (https://www.python.org/downloads/release/python-3104/) were used to create this figure.

For rodent animals, housing in an enriched environment (EE) has been shown to exert positive effects on various behavioral patterns and sleep quality^26,27^. Here, we examined whether living in an EE could restore aging-related LFP changes (Fig. 4A). First, we tested whether our experimental EE conditions were sufficient to affect memory performance. An object location test was utilized in which a mouse was first introduced to two identical objects in an open field (acquisition phase) and then exposed to the same two objects, one of which was displaced to a new location (test phase) (Fig. 4B). If the mouse remembered the locations of the objects in the acquisition phase, it would spend more time exploring the object in the new position. In this behavioral test, we combined datasets from a subset of the mice that were used for electrophysiological recordings and a subset of new mice that were not used for electrophysiological recordings (for more detail, see Materials and Methods). The location index, representing the interaction time with the novel object in the test phase, was significantly higher than the chance level (50%) in 10-week mice (*n* = 6 mice; *Z* = 2.20, *P* = 0.028, Wilcoxon signed rank test) but not in 2-year mice without EE (termed 2-year non-EE mice;* n* = 9 mice; *Z* = 0.18, *P* = 0.86, Wilcoxon signed rank test) (Fig. 4B). On the other hand, 2-year mice housed in EE (termed 2-year EE mice) for more than 2 weeks exhibited a location index that was significantly higher than chance (*n* = 9 mice; *Z* = 2.00, *P* = 0.036, Wilcoxon signed rank test). These results confirmed that the 2-year mice housed in EE had sufficient memory that could be detected by the object location test, similar to the 10-week mice, which were not observed in the 2-year mice without EE. Furthermore, the percentage of sleep time in 2-year EE mice was significantly lower than that in 2-year non-EE mice (Fig. 4C; *n* = 8 and 6 mice; *Z* = 2.65, *P* = 0.0081, Mann–Whitney U test).

Then we confirmed gene expression patterns in the dorsal hippocampus in these mouse groups. Aged mice exhibited altered expression patterns of a subset of genes related to neuron development and synaptic signaling (Supplementary Figs. 3A,C and 4). On the other hand, the 2-year EE mice exhibited increased or decreased expression levels of a subset of genes (as marked in blue or green in Supplementary Figs. 3A and 4), which became more similar to those observed from the 10-week-mice, confirming altered expression patterns of aging-related genes by EE.

Next, we applied the same analytical procedures as in Fig. 3F, plotting on UMAP dimensions and computing F1 scores, to the LFP datasets in all the frequency bands from the 2-year EE mice (Fig. 4D). Out of 6 mice tested, 5 (83.3%) mice exhibited significant F1 scores in the 2-year EE group, which was prominently higher than that (12.5% (1/8)) observed from the 2-year non-EE group (Fig. 4E). These results suggest that aged mice housed in EE conditions show brain network LFP patterns that distinguish awake/sleep states, which were not observed in the majority of the aged mice without EE conditions.

## Discussion

To understand senescence-induced changes in neuronal activity at the network level, we developed a device that simultaneously records local field potential signals from multiple brain regions in the forebrain together with EMG signals by improving our previous methods^28–30^. We confirmed that the total sleep time was not different between young and aged mice. While some studies have shown that sleep time becomes longer in aged mice^19,31^, this discrepancy may be due to experimental conditions such as recording time in a day or manually defined parameters to detect sleep periods^19–21,31,32^. We demonstrated that single regional correlations of LFP power changes (i.e. functional connections) computed in a single frequency band do not perfectly represent awake/sleep states in both of the ages. Notably, machine learning algorithms with UMAP and RCC applied to multidimensional datasets consisting of LFP patterns at multiple frequency bands together, ranging from delta to high-gamma bands, revealed that they could differentiate awake/sleep states in young mice, suggesting the usefulness of these analytical methods for awake/sleep-related LFP pattern analyses. We note that our recording was acute and restricted to 3.5 h with a single day in each animal. Longer or chronic recordings might provide further insights into how awake/sleep states undergo changes across days.

The importance of focusing on interregional activity patterns is consistent with the suggestions that awake and sleep brain states are not simply sustained by activity levels of single brain regions but generated from complex interactions across widespread brain regions^33,34^. When the datasets in all six frequency bands were combined, the representations of awake/sleep states by LFP signals were improved in young mice. On the other hand, these LFP correlational patterns less differentiated awake/sleep states in aged mice. The aging-induced changes in neuronal dynamics at the network levels may be explained by cooperative interactions among a number of molecular and cellular factors that have been reported to alter with aging, including decreases in neuromodulators^5^, reduced expression of neuromodulator receptors^35^, and decreased synchronicity of neurons in the suprachiasmatic nucleus^36^. Further studies are needed to determine which these microscopic mechanisms most strongly contribute to awake/sleep-related LFP patterns.

Finally, our results demonstrated that housing aged mice in EE conditions reinstated the aging-related decreases in LFP patterns such that they more precisely represented awake/sleep states. In addition to the positive effects of EE on various behavioral patterns^26,27^ and gene expression patterns^37,38^ reported previously, our study verified that EE can restore awake/sleep-related neuronal activity patterns. Considering that declines in sleep quality in senescence are crucial risk factors for neurodegenerative and psychiatric diseases^4^, EE is expected to prevent these pathological symptoms through improvements in the quality of sleep and sleep-related neuronal activity.

## Materials and methods

### Ethical approvals

Animal experiments were performed with the approval of the Animal Experiment Ethics Committee at The University of Tokyo (approval number: P29-14) and according to the ARRIVE guidelines and the University of Tokyo guidelines for the care and use of laboratory animals. These experimental protocols were carried out in accordance with the Fundamental Guidelines for Proper Conduct of Animal Experiment and Related Activities in Academic Research Institutions (Ministry of Education, Culture, Sports, Science and Technology, Notice No. 71 of 2006), the Standards for Breeding and Housing of and Pain Alleviation for Experimental Animals (Ministry of the Environment, Notice No. 88 of 2006) and the Guidelines on the Method of Animal Disposal (Prime Minister's Office, Notice No. 40 of 1995). All efforts were made to minimize the animals’ suffering.

### Animals

Male 8-to-10-week-old C57BL/6 mice (SLC, Shizuoka, Japan) and male 95-to-105-week-old C57BL/6 mice (Charles River Japan, Kanagawa, Japan) with a preoperative weight of 30 g were housed under conditions of controlled temperature and humidity (22 ± 1 °C, 55 ± 5%) in a vivarium, maintained on a 12:12-h light/dark cycle (lights off from 7 am to 7 pm) with ad libitum access to food and water. All mice were housed individually.

The numbers of mice used for electrophysiological recordings were seven (10-week), eight (2-year (non-EE)), and six (2-year (EE)). Of these recorded mice, four (10-week), five (2-year (non-EE)), and six (2-year (EE)) mice were used for the object location test. In addition, two (10-week), four (2-year (non-EE)), and three (2-year (EE)) mice were used for the object location test alone without electrophysiological recordings. No animals were excluded from our analyses after obtaining datasets.

### Enriched environment

All young 10-week-old mice were housed in a homecage. Two-year-old mice were randomly selected and exposed to either standard environment (non-EE) or enriched environment (EE) conditions for more than 2 weeks. For non-EE conditions, standard laboratory cages were used (24 cm × 17 cm × 12 cm), whereas mice maintained under EE conditions were housed in a rat cage (42 cm × 26 cm × 20 cm). The EE cages were equipped with a running wheel, a seesaw, a ball, and a climbing platform (Fig. 4A).

### Object location test

The experimental apparatus used in this study was an open-field box (47 cm × 37 cm with walls with a height of 31 cm). The apparatus was placed in a sound-isolated room. Identical plastic cones (10 cm in height × 6 cm in diameter) created using a 3D printer (Form 2, Formlabs, MA) were used as objects. This test consisted of a habituation day and a test day. On a habituation day, the mice were allowed to freely explore the apparatus without objects for 1 h. On the next day, which was the test day, the same habituation procedure was first conducted for 30 min before starting the acquisition phase. In the acquisition phase, the mice were allowed to freely explore two identical objects that were placed symmetrically in the experimental apparatus for 5 min. The mouse was then removed from the apparatus and returned to its home cage. The objects were thoroughly cleaned with 70% ethanol. The open field box was cleaned with dry paper after each trial to ensure that it was saturated with the smell of the animals. A test phase was conducted 1 h after the acquisition phase. In the test phase, one of the objects (A2) was moved to a different location (A2′(D)), and the other object (A1) remained in the same position (A1(ND)) as in the acquisition phase. The terms D and ND indicate the displaced and the nondisplaced objects in the test phase, respectively. In the test phase, the mouse was allowed to freely explore the experimental apparatus for 5 min. Their behavior was recorded with a video camera mounted above the apparatus. The time spent exploring each object was measured using DeepLabCut^39^. Exploration of an object was defined as pointing the nose toward the object at a distance of < 1 cm and/or touching it with the nose. To analyze memory performance, a location index was calculated as follows: TA2′(D)/(TA1(ND) + TA2′(D)), where TA2′(D) is the time exploring the displaced object and TA1(ND) is the time exploring the nondisplaced object.

### Preparation of an electrode array

The electrode array used in this study has been described elesewhere^40^. An electrode array for brain LFP recording was assembled consisting of custom-made parts (Fig. 1A) and an electrical interface board (EIB) (Neuralynx, Bozeman, MT). A plastic core body, created by a 3D printer, contained multiple small holes with a dimeter of 0.7 mm distributed in space corresponding with the XY-coordinates of the targeted cortical areas, which served as a template to determine the locations of electrodes. An electrode assembly was created by setting metal tubes and electrodes into these holes so that the tips of the electrodes corresponded with the depth of individual brain regions. The other open ends of the electrodes were connected to metal holes of an EIB mounted on the top of a core body. An EIB consisted of 30 LFP channels and 2 ground channels, and all electrical signals from these channels were transferred to an Omentins connector. For brain LFP recording, a nichrome wire (A-M Systems, WA) or a tetrode that was constructed by bundling together four 17-μm polyimide-coated platinum–iridium (90/10%) wires (California Fine Wire, CA) and plated with platinum used to adjust the electrode impedances to 150–300 kΩ were used. The size of the electrode assembly was width 20 mm, length 20 mm, height 41 mm, and weight 2.2 g. The open edges of all electrodes were soldered to the corresponding channels on the EIB.

### Surgery

Standard surgical procedures were similar to those described previously^28,41,42^. Animals were anesthetized with 1–2% of isoflurane gas in air. The animal was then fixed in a stereotaxic instrument with two ear bars and a nose clamp. First, two craniotomies were made; one covering the coordinates for the anterior cingulate cortex (ACC; 1.2 mm anterior and 0.2 mm lateral to the bregma), primary motor cortex (M1: 1.2 mm anterior and 1.6 mm lateral to the bregma), and the striatum (STR: 1.2 mm anterior and 1.6 mm lateral to the bregma), and the other covering the coordinates for primary somatosensory cortex (S1; 1.9 mm posterior and 3.0 mm lateral to the bregma), the hippocampus (HPC; 1.9 mm posterior and 1.4 mm lateral to the bregma), and the medial parietal association cortex (MPtA; 1.9 mm posterior and 0.5 mm lateral to the bregma). The electrode array was directly implanted into the cortical tissue in the right hemisphere with electrodes inserted 0.5 mm into S1 and MPtA, 1.0 mm into ACC and M1, 1.4 mm into HPC, and 2.3 mm into STR. An electromyogram (EMG) electrode was implanted into the dorsal neck area. For the cerebellum, stainless steel screws were implanted on the skull attached to the brain surface to serve as ground/reference electrodes. Finally, all of the wires and the electrode array were secured to the skull using dental cement. After completing all surgical procedures, the anesthesia was terminated and the animals were spontaneously allowed to awake from the anesthesia. Following surgery, each animal was housed in a standard environment or an enriched environment with free access to water and food, with daily observation.

### In vivo* electrophysiology*

For electrophysiological recording, the animals were kept in their home cage and the homecage accommodating the mice were transported from the vivarium to a soundproof room. For the mice in the EE condition, all of the objects (e.g. the running wheel, the seesaw, the ball, and the climbing platform) were removed from the cage so that the recording conditions were consistent across all mouse groups. After transporting into the recording room, recordings started within 30 min. Under this condition, the mice were already acclimated to local environments in the homecage while they to some extent recognized the novelty of distal environments of the room. The EIB on the animal's head was connected to a digital headstage Cereplex M and Cereplex μ (Blackrock Microsystems), and signals were digitized and they were transferred to an electrophysiological data acquisition system Cereplex Direct (Blackrock Microsystems). Local field potential (LFP) recordings commenced at a sampling rate of 2 kHz for 2.5–3.5 h. For all recordings, electrophysiological signals were filtered between 0.1 Hz and 500 Hz. All electrophysiological recordings were performed between 10 am and 2 pm.

### Histology of brain tissue

The procedure has been described elesewhere^29,43^. After the recordings, the mice were perfused intracardially with cold 4% paraformaldehyde (PFA) in 25 mM phosphate-buffered saline (PBS) and decapitated. The electrodes were carefully removed from the brain 6–8 h after the perfusion. The brains were placed in 30% sucrose until equilibrated and coronally sectioned at a thickness of 50 mm, and the slices were stained with cresyl violet.

### Definition of awake/sleep states

All analyses were performed using custom-made MATLAB 2020a (MathWorks, MA, USA) and Python 3 routines. The awake/sleep states were determined using the same algorithm as in the previous study^19^. Briefly, active awake states were defined first based on EMG signals. The root mean square (RMS) of the EMG signals was computed every 1 s with a window of 5 s. Active awake states were defined as periods when EMG RMS was above a threshold (Supplementary Fig. 1C, top raw). The threshold was manually adjusted for individual mice with the aid of visual inspection of EMG RMS traces^19^. The threshold was the mean + 0.8–1.2 × SD, which corresponded with an animal’s moving speed of 3 cm/s (Supplementary Fig. 1B), a speed generally considered to fully represent mouse’s movement, not optical noise during sleep states. Periods with EMG RMS of the mean + 5.0 × SD was considered as massive noise and excluded from the analysis. For the other periods except active awake states, we computed the ratio of delta power to theta power in cortical LFP signals every 1 s with a window of 5 s and slow wave sleep (SWS) states were defined as periods when EMG RMS was above a threshold (Supplementary Fig. 1C, middle raw). The threshold was manually adjusted for individual mice with the aid of visual inspection of LFP power traces^20,21^. The threshold was the mean + 0.2–0.5 × SD. For the other periods except active awake and SWS states, we computed an index, LFP theta power/(LFP delta power × EMG RMS), which has been utilized as an efficient measure to detect rapid eye movement (REM) sleep states^19^, every 1 s with a window of 5 s, and REM sleep states were defined as periods when this index was above a threshold (Supplementary Fig. 1C, bottom raw). The threshold was manually adjusted for individual mice with the aid of visual inspection of LFP power traces^19^. The threshold was the mean + 1.0–1.2 × SD. The other periods that were not classified into these three states were classified into quiet awake periods. Active awake and quiet awake states were considered as awake periods, whereas SWS and REM sleep states were considered as sleep periods.

### LFP power and correlation analysis

LFP signals were downsampled to 400 Hz, and LFP spectral power in six frequency bands (δ: 1–4 Hz, θ: 4–8 Hz, α: 8–13 Hz, β: 13–30 Hz, low-γ: 30–50 Hz, and high-γ: 50–100 Hz) was computed by Morlet wavelet analysis. Similar to the definition of awake/sleep states, the RMS of the EMG signals was computed every 1 s. For LFP analysis, LFP signals from 2.5–3.5 h were divided into 10-s bins. An awake bin was defined when a 10-s bin contained awake time shorter than 5 s, whereas a sleep bin was defined when a 10-s bin contained sleep time of 5 s or longer than 5 s. In each bin, correlations of LFP power changes in each frequency band were computed from the 15 pairs of brain regions to obtain a 15-dimensional vector. When specified, vectors in all five frequency bands were combined to obtain a 90-dimensional vector.

### Machine learning algorithms

Uniform manifold approximation and projection (UMAP) was used for nonlinear dimensionality reduction^44^. With UMAP, 15- or 90-dimensional vectors were reduced to two dimensions with the following hyperparameters: *n_neighbors* = 3, *min_dist* = 0.1, *n_components* = 2, and *metric* = ‘euclidean'. We verified that “*n_neighbors*” ranging from 3 to 5 and “*min_dist*” ranging from 0 to 0.1 yielded the same statistical results. Then, a robust continuous clustering (RCC) algorithm, an unsupervised clustering method, was applied to the plots from UMAP (Python implemented with the following hyperparameters: *clustering_threshold* = 60, *k* = 60, and *measure* = ‘euclidean’) (Fig. 3)^45^. We verified that “*clustering_threshold*” ranging from 40 to 80 and “*k*” ranging from 40 to 80 yielded the same statistical results.

If more than half of the bins in a cluster defined by the RCC were awake or sleep bins, the cluster was classified as an awake cluster or a sleep cluster, respectively. To quantify the degree of the separation between awake/sleep bins in awake/sleep clusters, in each animal, F1 scores for awake and sleep bins (termed F1_awake_ and F1_sleep_, respectively) were computed as the harmonic mean of precision and recall, where precision was the ratio of the number of awake/sleep bins included in awake/sleep clusters to the number of all bins included in the awake/sleep cluster, and recall was the ratio of the number of awake/sleep bins included in awake/sleep clusters to the number of all awake/sleep bins. In each animal, an F1 score was computed as (F1_awake_ + F1_sleep_)/2. To assess the significance of an F1 score, shuffled datasets were constructed for each original UMAP plot by randomly shuffling bin types (awake/sleep) assigned to the plots. For more limited conditions, shuffling was performed every 18 consecutive bins (*i.e.* 180 s), a duration representing the majority of sleep duration, which could keep temporal structures of the order and vicinity of epochs within this time period. An F1 score for the original data was considered to be significant when it was higher than top 5% of F1 scores computed from 1000 shuffled datasets.

### Gene expression analysis

For gene expression analysis, tissue samples were obtained from the dorsal hippocampus. Total RNA was extracted from the tissue using the RNeasy Plus Mini Kit (Qiagen Inc., Valencia, CA, USA). Microarray studies were performed using Affymetrix GeneChip Mouse Clariom S arrays (Thermo Fisher Scientific, Hampton, NH, USA). Gene expression analysis was performed using iDEP (integrated Differential Expression and Pathway analysis; Steven, Runan, 2018, BMC Bioinformatics).

### Statistics

All data are presented as the mean ± standard error of the mean (SEM), unless otherwise specified, and were analyzed using Python and MATLAB. Comparisons of two-sample data were analyzed by Wilcoxon signed rank test for matched samples and Mann–Whitney U test for independent samples. Multiple group comparisons were performed by post hoc Bonferroni corrections. The null hypothesis was rejected at the *P* < 0.05 level.

## Supplementary Information

Supplementary Information.

## Data Availability

The datasets collected and analyzed are available from the corresponding author on request.

## References

[1] Bliwise DL (1993). Sleep in normal aging and dementia. Sleep.

[2] Achermann P, Borbely AA (1997). Low-frequency (< 1 Hz) oscillations in the human sleep electroencephalogram. Neuroscience.

[3] Campos-Beltran D, Marshall L (2021). Changes in sleep EEG with aging in humans and rodents. Pflugers Arch..

[4] Bjorvatn B (2007). The association between sleep duration, body mass index and metabolic measures in the Hordaland Health Study. J. Sleep Res..

[5] Utkin YN (2019). Aging affects nicotinic acetylcholine receptors in brain. Cent. Nerv. Syst. Agents Med. Chem..

[6] Morrison JH, Hof PR (1997). Life and death of neurons in the aging brain. Science.

[7] Morrison JH, Hof PR (2007). Life and death of neurons in the aging cerebral cortex. Int. Rev. Neurobiol..

[8] Andrews-Hanna JR (2007). Disruption of large-scale brain systems in advanced aging. Neuron.

[9] Resnick SM, Pham DL, Kraut MA, Zonderman AB, Davatzikos C (2003). Longitudinal magnetic resonance imaging studies of older adults: A shrinking brain. J. Neurosci..

[10] Svennerholm L, Bostrom K, Jungbjer B (1997). Changes in weight and compositions of major membrane components of human brain during the span of adult human life of Swedes. Acta Neuropathol..

[11] Hedden T, Gabrieli JD (2004). Insights into the ageing mind: A view from cognitive neuroscience. Nat. Rev. Neurosci..

[12] Bartzokis G (2003). White matter structural integrity in healthy aging adults and patients with Alzheimer disease: A magnetic resonance imaging study. Arch. Neurol..

[13] Carrier J (2011). Sleep slow wave changes during the middle years of life. Eur. J. Neurosci..

[14] Mander BA, Winer JR, Walker MP (2017). Sleep and human aging. Neuron.

[15] Murty D (2020). Gamma oscillations weaken with age in healthy elderly in human EEG. Neuroimage.

[16] Wimmer ME (2013). Aging in mice reduces the ability to sustain sleep/wake states. PLoS ONE.

[17] Brown RE, Basheer R, McKenna JT, Strecker RE, McCarley RW (2012). Control of sleep and wakefulness. Physiol. Rev..

[18] Yuan R (2012). Genetic coregulation of age of female sexual maturation and lifespan through circulating IGF1 among inbred mouse strains. Proc. Natl. Acad. Sci. U. S. A..

[19] Soltani S (2019). Sleep-wake cycle in young and older mice. Front. Syst. Neurosci..

[20] Grosmark AD, Mizuseki K, Pastalkova E, Diba K, Buzsaki G (2012). REM sleep reorganizes hippocampal excitability. Neuron.

[21] Mizuseki K, Diba K, Pastalkova E, Buzsaki G (2011). Hippocampal CA1 pyramidal cells form functionally distinct sublayers. Nat. Neurosci..

[22] Meng Q (2021). Tracking eye movements during sleep in mice. Front. Neurosci..

[23] Libourel PA, Corneyllie A, Luppi PH, Chouvet G, Gervasoni D (2015). Unsupervised online classifier in sleep scoring for sleep deprivation studies. Sleep.

[24] Gervasoni D (2004). Global forebrain dynamics predict rat behavioral states and their transitions. J. Neurosci..

[25] Kaminski M, Blinowska K, Szclenberger W (1997). Topographic analysis of coherence and propagation of EEG activity during sleep and wakefulness. Electroencephalogr. Clin. Neurophysiol..

[26] Garthe A, Roeder I, Kempermann G (2016). Mice in an enriched environment learn more flexibly because of adult hippocampal neurogenesis. Hippocampus.

[27] van Gool WA, Mirmiran M (1986). Effects of aging and housing in an enriched environment on sleep-wake patterns in rats. Sleep.

[28] Konno D (2019). Collection of biochemical samples with brain-wide electrophysiological recordings from a freely moving rodent. J. Pharmacol. Sci..

[29] Okada S, Igata H, Sakaguchi T, Sasaki T, Ikegaya Y (2016). A new device for the simultaneous recording of cerebral, cardiac, and muscular electrical activity in freely moving rodents. J. Pharmacol. Sci..

[30] Nakayama R, Ikegaya Y, Sasaki T (2019). Cortical-wide functional correlations are associated with stress-induced cardiac dysfunctions in individual rats. Sci. Rep..

[31] McKillop LE (2018). Effects of aging on cortical neural dynamics and local sleep homeostasis in mice. J. Neurosci..

[32] Hasan S, Dauvilliers Y, Mongrain V, Franken P, Tafti M (2012). Age-related changes in sleep in inbred mice are genotype dependent. Neurobiol. Aging.

[33] Scammell TE, Arrigoni E, Lipton JO (2017). Neural circuitry of wakefulness and sleep. Neuron.

[34] Eban-Rothschild A, Appelbaum L, de Lecea L (2018). Neuronal mechanisms for sleep/wake regulation and modulatory drive. Neuropsychopharmacology.

[35] McEntee WJ, Crook TH (1991). Serotonin, memory, and the aging brain. Psychopharmacology.

[36] Farajnia S, Deboer T, Rohling JH, Meijer JH, Michel S (2014). Aging of the suprachiasmatic clock. Neuroscientist.

[37] Wang X (2020). Enriched environment enhances histone acetylation of NMDA receptor in the hippocampus and improves cognitive dysfunction in aged mice. Neural Regen. Res..

[38] Dong BE, Chen H, Sakata K (2020). BDNF deficiency and enriched environment treatment affect neurotransmitter gene expression differently across ages. J. Neurochem..

[39] Mathis A (2018). DeepLabCut: Markerless pose estimation of user-defined body parts with deep learning. Nat. Neurosci..

[40] Okonogi T, Sasaki T (2021). Theta-range oscillations in stress-induced mental disorders as an oscillotherapeutic target. Front. Behav. Neurosci..

[41] Sasaki T, Nishimura Y, Ikegaya Y (2017). Simultaneous recordings of central and peripheral bioelectrical signals in a freely moving rodent. Biol. Pharm. Bull..

[42] Shikano Y, Sasaki T, Ikegaya Y (2018). Simultaneous recordings of cortical local field potentials, electrocardiogram, electromyogram, and breathing rhythm from a freely moving rat. J. Vis. Exp..

[43] Aoki Y (2017). Selective attenuation of electrophysiological activity of the dentate gyrus in a social defeat mouse model. J. Physiol. Sci..

[44] McInnes, L., Healy, J. & Melville, J. UMAP: Uniform Manifold Approximation and Projection for Dimension Reduction. arXiv:1802.03426 (2018).

[45] Shah SA, Koltun V (2017). Robust continuous clustering. Proc. Natl. Acad. Sci. U.S.A..

