# Supplementary material for: Weak representation of awake/sleep states by local field potentials in aged mice
Source: Sci Rep. 2022 May 11;12:7766. doi: 10.1038/s41598-022-11888-0 (PMC9095686; doi:10.1038/s41598-022-11888-0)
Supplement: Supplementary file 1 — Supplementary Information. [file 41598_2022_11888_MOESM1_ESM.docx]

*Scientific Reports*

# Supplementary Materials for

# Weak representation of awake/sleep states

# by local field potentials in aged mice

Daichi Konno, Yuji Ikegaya, Takuya Sasaki^†^

† To whom correspondence should be addressed:

Takuya Sasaki, takuya.sasaki.b4@tohoku.ac.jp

This file includes:

Figs. S1 to S4

# Figure Legends

#
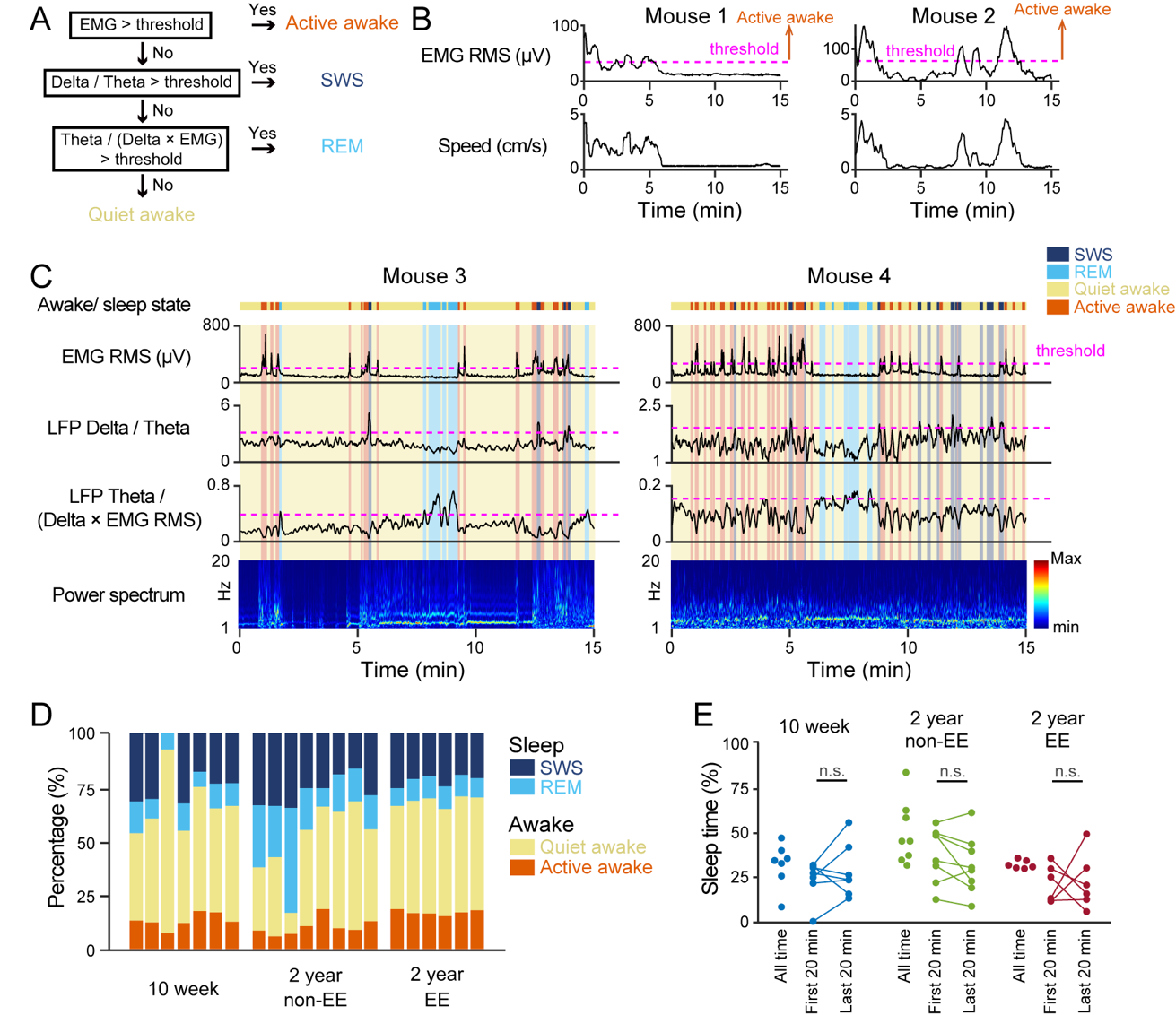


## **Figure S1 Definition of awake/sleep states based on EMG and LFP signals**. (A) A chart of algorithm for determining each state. (B) Representative two mice showing temporal changes in EMG RMS and moving speed monitored by a video camera. Note that the two variables show nearly similar patterns. The horizontal dotted lines represent a threshold. Periods with EMG RMS exceeding the threshold were defined as active awake states. (B) Two representative mice are shown. (From top to bottom) Time changes in EMG RMS, LFP delta/theta ratio, LFP theta/(delta × EMG RMS), and LFP power spectrum. Each horizontal dotted line represents a threshold. Awake/sleep states defined from these physiological signals are represented as colored bars. (C) Proportions of awake/sleep states in individual animals (*n* = 7, 8 and 6 mice). (D) In each animal group, proportions of sleep time in all recording time (left plots) and the comparisons between the first and last 20 min of the recording time (right two plots connected by lines). Each plot shows each mouse. MATLAB R2021b (https://jp.mathworks.com/products/new_products/release2021b.html) and Illustrator 26.2.1 (https://www.adobe.com/jp/products/illustrator.html) were used to create this figure.

##
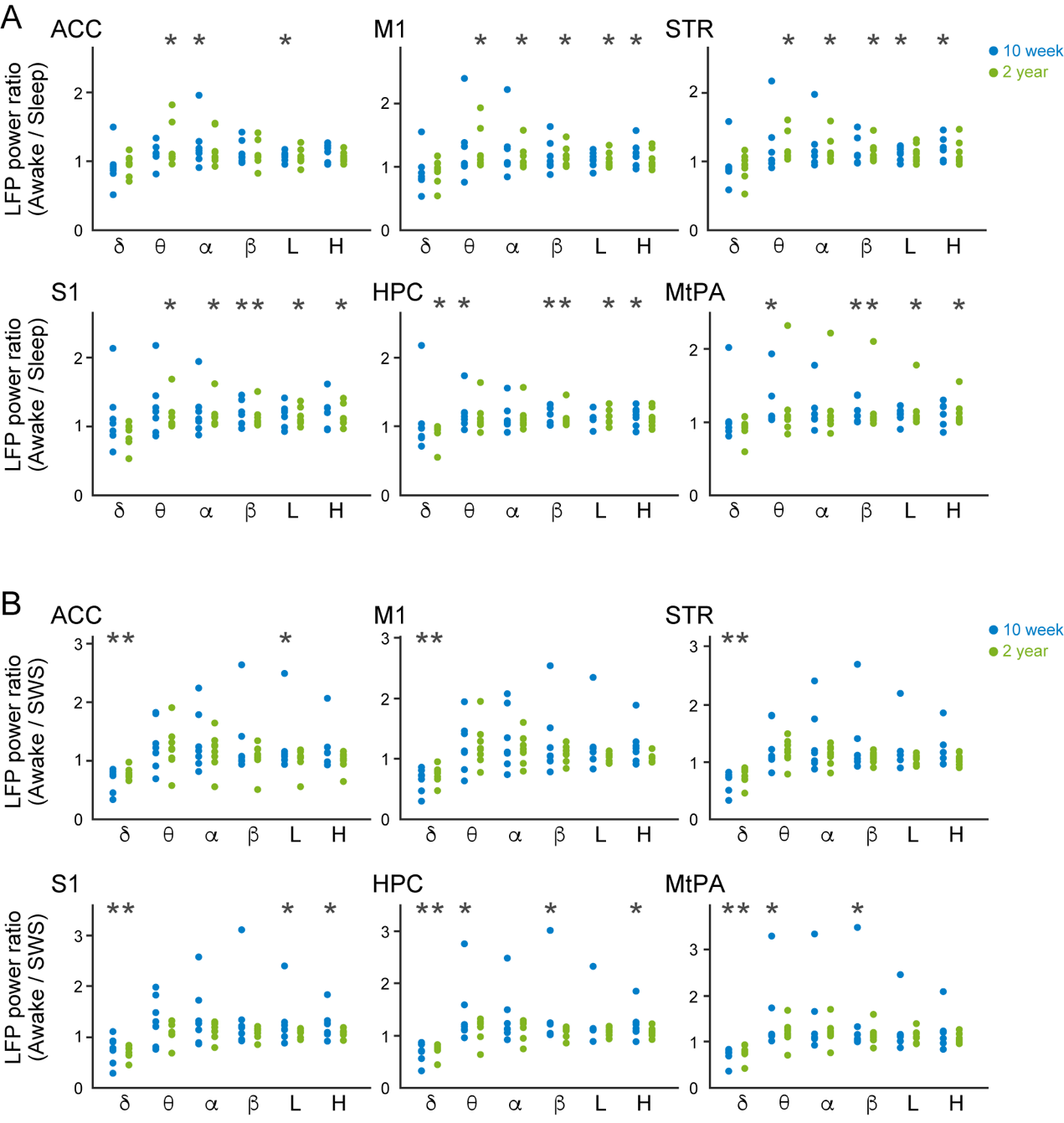
**Figure S2 LFP power in single frequency bands in single brain regions in awake and sleep states.** (A) LFP power in individual frequency bands in individual brain regions in awake states was normalized by the average in entire sleep states (*n* = 7 and 8 mice). **P* < 0.05, Wilcoxon signed rank test versus 1 in each group. (B) Same as A but normalized by the average in SWS states. MATLAB R2021b (https://jp.mathworks.com/products/new_products/release2021b.html) and Illustrator 26.2.1 (https://www.adobe.com/jp/products/illustrator.html) were used to create this figure.

##
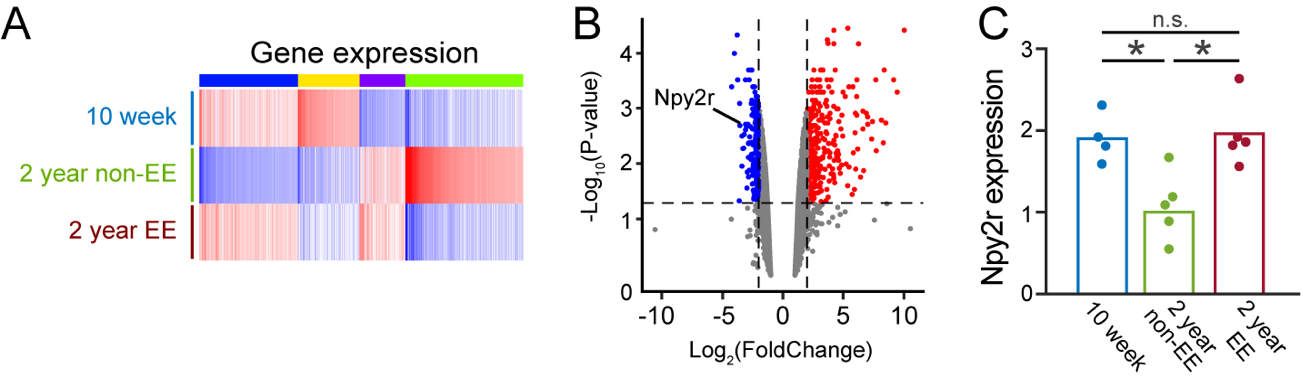
**Figure S3 Gene expression in 2-year EE mice. (A)** Heatmaps of representative gene expression patterns in the dorsal hippocampus of 10-week, 2-year non-EE, and 2-year EE mouse groups. Of the 21200 genes, the top 2000 genes with large expression variations between groups are shown. The top color bar shows clusters defined by the *K*-means algorithm. The gene ontology of the genes in each cluster is shown in Figure S4. **(B)** Volcano plot showing the fold change and *P* value of individual genes in 2-year non-EE mice compared with 10-week mice. The vertical dotted lines indicate 2-fold upregulation and downregulation, and the horizontal dotted line represents the cutoff significance level (*P* = 0.05). The red and blue dots indicate significantly upregulated and downregulated genes, respectively, in aged mice. **(C)** qPCR analysis validating the expression levels of Npy2r, a gene found to be differentially expressed in the bulk RNA-seq analysis across the mouse groups (*n* = 4, 5, and 5 mice). Each dot represents an individual mouse. **P* < 0.05, Student's *t*-test. MATLAB R2021b (https://jp.mathworks.com/products/new_products/release2021b.html), Illustrator 26.2.1 (https://www.adobe.com/jp/products/illustrator.html), Python 3.10.4 (https://www.python.org/downloads/release/python-3104/) and iDEP.95 (http://bioinformatics.sdstate.edu/idep95/) were used to create this figure.

##
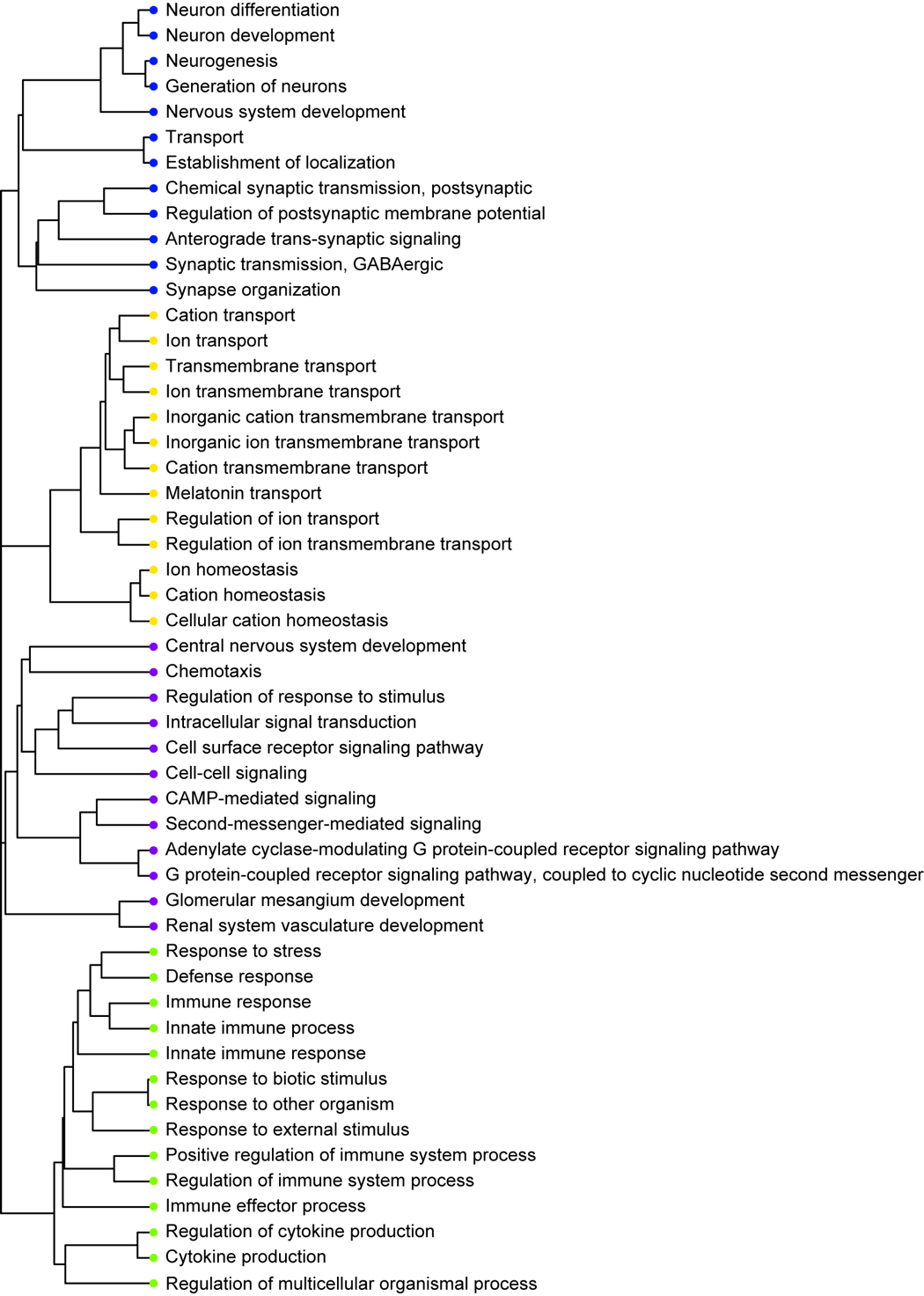
**Figure S4, Gene Ontology (GO) enrichment analysis for each cluster of the heatmap shown in Figure S2A.** The cluster with increased expression in the 2-year non-EE group (green) contains GO terms related to stress and immune responses, while the cluster with increased expression in the 10-week and 2-year EE groups (blue) contains GO terms related to neurogenesis and synaptic transmission. iDEP.95 (http://bioinformatics.sdstate.edu/idep95/) was used to create this figure.
